# A Cocktail of Thermally Stable, Chemically Synthesized Capture Agents for the Efficient Detection of Anti-Gp41 Antibodies from Human Sera

**DOI:** 10.1371/journal.pone.0076224

**Published:** 2013-10-07

**Authors:** Jessica A. Pfeilsticker, Aiko Umeda, Blake Farrow, Connie L. Hsueh, Kaycie M. Deyle, Jocelyn T. Kim, Bert T. Lai, James R. Heath

**Affiliations:** 1 Division of Chemistry and Chemical Engineering, California Institute of Technology, Pasadena, California, United States of America; 2 Division of Engineering and Applied Sciences, Division of Biology, California Institute of Technology, Pasadena, California, United States of America; 3 Division of Biology, California Institute of Technology, Pasadena, California, United States of America; 4 Indi Molecular, Culver City, California, United States of America; New York University, United States of America

## Abstract

We report on a method to improve *in vitro* diagnostic assays that detect immune response, with specific application to HIV-1. The inherent polyclonal diversity of the humoral immune response was addressed by using sequential *in situ* click chemistry to develop a cocktail of peptide-based capture agents, the components of which were raised against different, representative anti-HIV antibodies that bind to a conserved epitope of the HIV-1 envelope protein gp41. The cocktail was used to detect anti-HIV-1 antibodies from a panel of sera collected from HIV-positive patients, with improved signal-to-noise ratio relative to the gold standard commercial recombinant protein antigen. The capture agents were stable when stored as a powder for two months at temperatures close to 60^o^C.

## Introduction

Detecting the immune response to an infectious agent can provide a useful *in vitro* diagnostic surrogate relative to direct pathogen detection [1]. Such assays are commonly used for detecting HIV infection because of its characteristic immunopathology [2]. Direct detection of HIV viral RNA and p24 antigen is only effective at an early stage of infection, approximately 2–6 weeks of initial exposure [3], [4]. Antibodies against HIV envelope proteins emerge in patients’ blood around 3–4 weeks of infection [2], [5] as the viral RNA and p24 levels decline as a result of immunocomplex formation [6]. The high serum level of anti-HIV IgG is maintained throughout the course of clinical latency (2–20+ years), during which time viral antigens are under detection limits until the onset of acquired immunodeficiency syndrome (AIDS) [2], [5]. Viral load and CD4^+^ cell counts are mainly used for prognostic purposes to monitor the efficacy of treatments; however viral load is sometimes used for the diagnosis of infant HIV infections where antibody-based assays are not applicable [3], [7]. Assays for anti-HIV antibodies are the most widely used diagnostic test both in cases where infection is presumed to have occurred more than 6 weeks prior to testing, and for epidemiological reasons, to estimate the incidence of HIV in a population [8], since, with the exception of infant HIV, virtually 100% of the infected individuals express these antibodies [3]. Typically in these assays, immunogenic and conserved antigens from the HIV are expressed as regions of a single chimeric protein. That chimeric protein is then used to capture specific antibodies from the body fluid (e.g. blood, saliva or urine) of potentially infected patients; a positive assay result implies infection. However, the polyclonal diversity of antibodies across a patient population can translate into large variations in assay performance from patient to patient. In addition, the chimeric recombinant proteins are biological reagents, and so may have limitations related to shelf life and batch-to-batch variability. These limitations can adversely influence the performance of a diagnostic test [3], [5], [9], especially one that is deployed in harsh physical environments.

Here we report on the use of iterative *in situ* click chemistry [10], [11] to prepare a cocktail of chemically synthesized capture agents (called protein-catalyzed capture agents, or PCC Agents) that is designed to sample the polyclonal diversity of an antibody-based immune response. We demonstrate the concept by developing a PCC Agent-based assay designed to detect human antibodies that bind to a conserved region of the HIV-1 envelope glycoprotein gp41. The performance of that assay is compared against the gold standard chimeric protein antigen using sera collected from a cohort of HIV-1-positive human subjects, plus controls. We also report on the thermal stability of the capture agent cocktail, with an eye towards point-of-care HIV diagnostics assays that are needed in environments where refrigeration chains may not exist.

## Materials and Methods

For detailed protocols see **Materials and Methods S1**.

### Ethics Statement

All study documents and procedures regarding the patient serum assays were approved by the UCLA and Caltech Institutional Review Boards. All subjects provided written informed consent prior to initiation of study procedures.

## Results and Discussion

The development of a PCC Agent against a protein target utilizes the target itself to promote the 1,3-dipolar cycloaddition between an acetylene and an azide group to form a triazole linkage (the *in situ* ‘click’ reaction) [12]. The protein effectively plays the role of an extremely selective, but much less efficient, variant of the Cu(I) catalyst that is commonly used for such couplings [13], [14]. For the present work, the two reacting species are peptides – one peptide (the anchor) is a chemically modified variant of a conserved, immunogenic epitope on the HIV-1 gp41 protein, and the second peptide is selected via an *in situ* click screen from a large (10^6^ element) one-bead-one-compound (OBOC) [15] peptide library. The protein targets are human monoclonal antibodies raised against variants of the gp41 epitope represented by the anchor peptide.

The PCC Agents developed here were designed to capture antibodies that are selective for residues 600–612 (IWCGSGKLICTTA) of gp41. Previous studies have shown that a large fraction of HIV-1-positive patients develop antibodies against this epitope [16], [17]. Our strategy for sampling the polyclonal diversity of such antibodies was to develop PCC Agents that exhibited both differential, as well as similar avidities for human monoclonal antibodies (mAbs) raised against different parts of this epitope. A key decision in this regard involves the selection of the anchor peptides from which the PCC Agents are developed. For this task, we modified the polypeptide fragment corresponding to residues 600–612 of gp41 with artificial amino acids at multiple locations, and tested the ability of these modified peptides to detect two different monoclonal anti-gp41 antibodies 3D6 and 4B3 (Polymun, Klosterneuburg, Austria). The 3D6 mAb was raised against the epitope SGKLIC, whereas the 4B3 mAb was raised against SGKLICTTA. One anchor peptide, A21, was synthesized by adding a propargyl glycine (Pra) residue at the C-terminus of the residues 600–612 of gp41. This anchor peptide was also N-terminally tagged with a polyethylene glycol (PEG) oligomer bridge and a biotin label. For a second anchor peptide (A22), Leu-607 was substituted with Pra. A22 also included an N-terminal PEG-biotin label. A21 equally detected 3D6 and 4B3 with an estimated dissociation constant (*K_d_*) of 1–50 nM, while A22 differentially detected 3D6 (*K_d_*>10 µM) and 4B3 (*K_d_* = 1–50 nM) (**Figure S1** in Supporting Information). A21 and A22 were then separately developed into PCC agents against 3D6 and 4B3, respectively.

**Figure 1:**
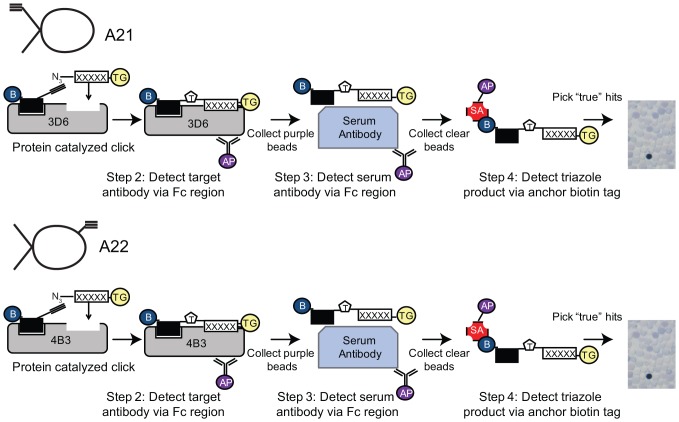
Screening strategy for selecting capture agents against anti-HIV antibodies 3D6 and 4B3. The flow chart represents the use of the A21 and A22 cyclic peptides as anchor ligands for separate in situ click screens against a large OBOC azide-presenting peptide library.

**Figure 2:**
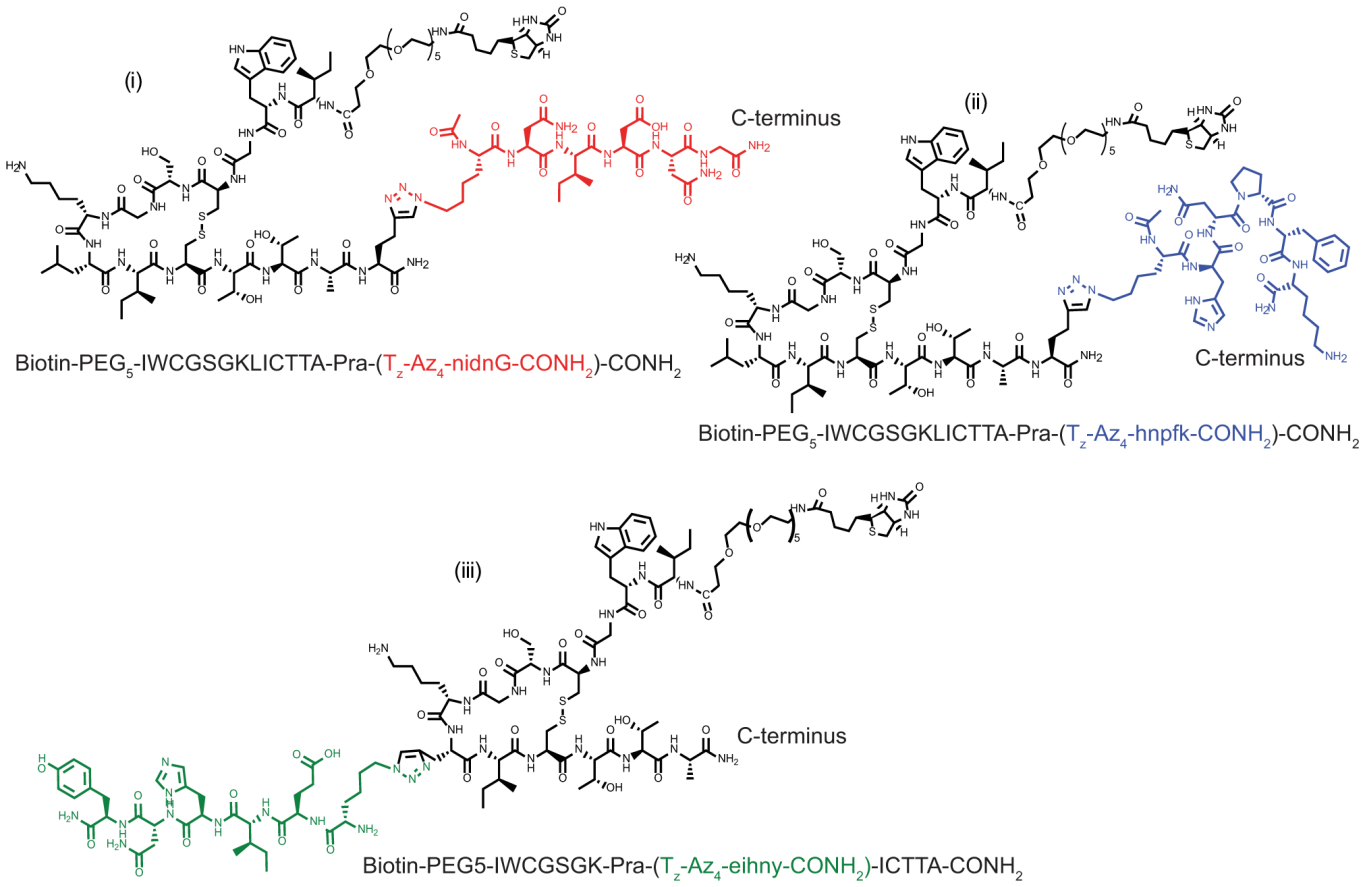
Structures of peptide ligands in PCC Agent cocktail. Acetylene-presenting anchor peptides (black) were derived from the immunogenic epitope of HIV-1 gp41 (residues 600–612). A22-nindG (**i**) and A21-hnpfk (**ii**) were evolved from the original epitope appended with Pra at the C-terminus whereas A22-eihny (**iii**) utilizes the “substituted” anchor where residue Leu-607 is replaced with Pra. Secondary ligand branches (colored) were identified from the *in situ* click screen of a 5-mer OBOC library presenting an azide functionality. Biligands (**i**) and (**ii**) were raised against the target anti-HIV antibody 3D6, and the biligand (**iii**) was raised against the antibody 4B3.

The *in situ* click screens are illustrated in **Figure 1**. The target IgG is incubated with an excess of the selected anchor peptide and a large OBOC library at 4^o^C overnight (see **Materials and Methods S1**). The OBOC library is synthesized on TentaGel resin (Rapp Polymere, Tuebingen, Germany), and is a comprehensive library of 5-mers with a 6^th^ amino acid at the N-terminus presenting an azide functionality. To help ensure chemical and biochemical stability, the OBOC library is comprised of non-natural (D) stereoisomers of the 20 natural amino acids, excluding cysteine and methionine. The *in situ* screen is designed to identify a secondary (2^o^) peptide that, when coupled to the anchor, forms a biligand with increased selectivity and/or affinity for the target IgG. The screen proceeds stepwise. In the first step (not shown in **Figure 1**) the OBOC library is cleared of beads that exhibit non-specific binding to alkaline phosphatase-conjugated streptavidin (SA-AP), which is used as a detection reagent in a later step. Step 2 is a target screen, and so is designed to detect the presence of the bound IgG target to specific beads, and defines possible hits. The step 3 screen is designed to remove those beads from the pool of potential hits that also exhibit binding to off-target serum proteins. Step 4 is called a product screen, and is unique to sequential *in situ* click screens [11]. This screen is designed to detect for the presence of *in situ* clicked reaction products, which are those hit beads containing the triazole-linked anchor peptide. Typically, Step 2 yields a few hundred hits (∼0.05% of the OBOC library). Step 3 reduces that pool by a factor of 2 or 3 to about 100 hits, and Step 4 further reduces the number of hits to around 10. This is a manageable number, meaning that each hit can be separately synthesized as a biligand using Cu(I) catalyzed click chemistry to couple the anchor and 2° peptides. The performance of these biligands is then characterized using immunoprecipitation (pulldown) assays detected by Western blotting from spiked serum samples for specificity (data not shown), and sandwich ELISAs and surface plasmon resonance (SPR) assays for affinity estimations (**Figure S2, Figure S3**). A complete list of the hits for the A21/3D6 and A22/4B3 screens is given in **Table S1**. This approach yielded two equivalently performing biligands against 3D6, and one biligand against 4B3 (**Figures S2, Figure S3**). These three PCC agents (**Figure 2**) were combined, in equal parts, to form a capture agent cocktail. The cocktail slightly outperformed both the standard commercial chimeric antigen and A21, when tested against healthy human serum spiked with both 3D6 and 4B3 (**Figure S4**). A21 is the equivalent of the original antigenic epitope of gp41.

**Figure 3:**
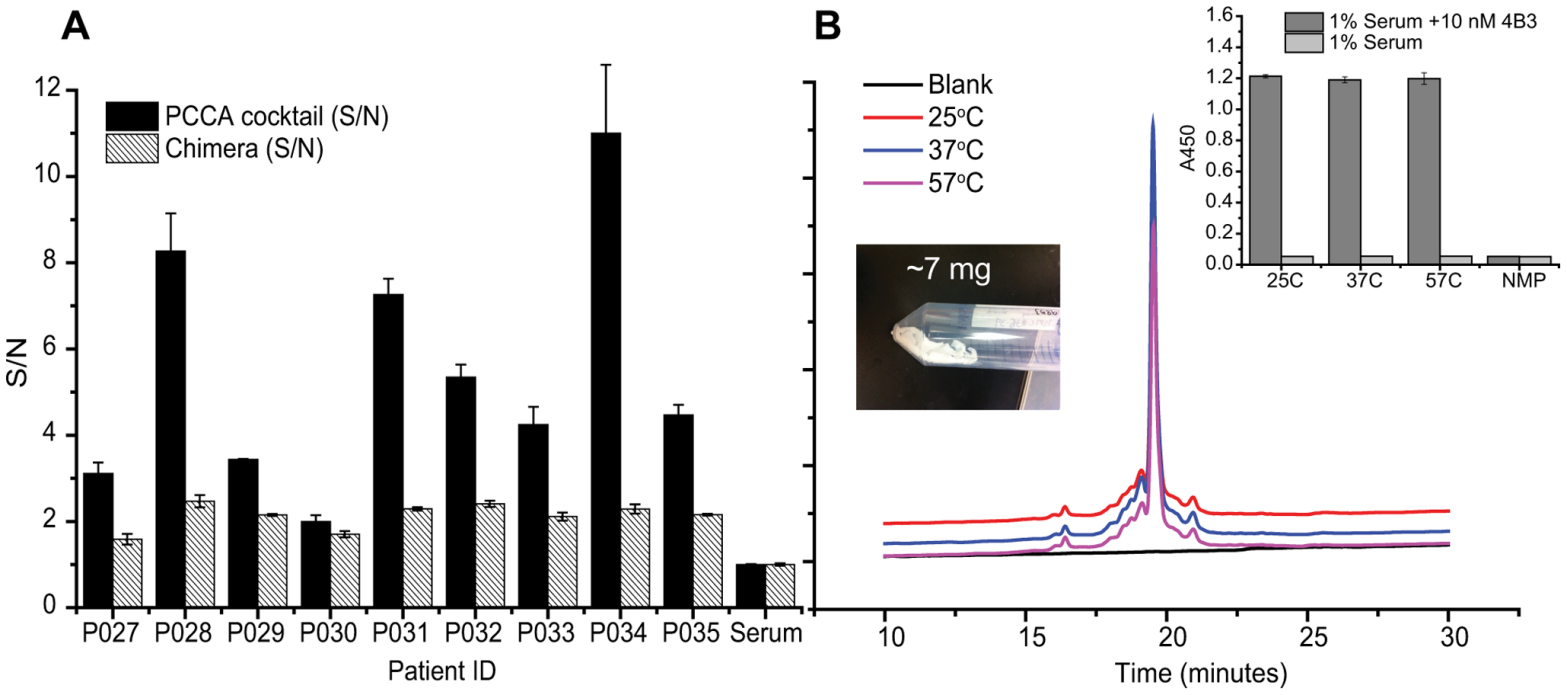
Performance of the PCC Agent cocktail, and thermal stability and scale up of a cocktail component. **A.** Comparative performance of the PCC Agent cocktail versus the commercial chimeric protein, using sandwich ELISAs to detect anti-HIV-1 IgGs from a panel of sera samples collected from nine HIV-positive patients. The absorbance at 450 nm (A450) for each sample is normalized against the A450 for the healthy control, to yield a measurement of the signal-to-noise ratio of the assay. The PCC Agent cocktail, which is designed to capture a subset of anti-gp41 IgGs, exhibits superior performance for every sample, even though the chimeric protein is designed to capture antibodies against multiple HIV-1-associated epitopes (those containing fragments of HIV-1 gp41, “O” group HIV-1 gp41 immunodominant region, and HIV-2 gp39). For the assays, the PCC agent cocktail and the biotinylated chimeric antigen were immobilized on a streptavidin-coated 96-well plate and incubated with diluted patient serum (1% v/v). Captured anti-HIV antibodies were detected using peroxidase-conjugated anti-human IgG antibody. **B.** Samples of **(iii)** were stored as a powder (inset photo), under N_2_ at temperatures up to 57°C for ∼2 months, and resolved by analytical HPLC to determine the presence of any degradation product. The HPLC traces reveal that the fingerprint of the PCC Agent is unchanged. The inset shows that the assay performance of the PCC Agent is also unaffected.

The PCC Agent cocktail and the standard antigen were co-evaluated against a panel of clinical samples using sandwich ELISAs. Serum samples were collected from nine HIV-1-positive patients in Southern California. The standard antigen is a recombinant chimeric protein containing a fragment of HIV-1 gp41 (residues 546–692), the “O” group HIV-1 gp41 immunodominant region (residues 580–623), and a fragment of HIV-2 gp36 (residues 591–617). A good performance of this chimeric antigen has been reported elsewhere [18]. For the comparison assays, streptavidin-coated 96-well plates were saturated with the PCC Agent cocktail or chemically biotinylated chimera in triplicates. Serum samples were diluted to 1% v/v in Tris-buffered saline (TBS) supplemented with 0.1% w/v bovine serum albumin (BSA), and incubated in the wells for 1 hr at room temperature. Unbound proteins were washed off and captured IgG was detected with a peroxidase-conjugated mouse monoclonal anti-human IgG-Fc antibody. The comparison assay results for the patient serum samples along with a healthy control are shown in **Figure 3A**. The signal-to-noise (S/N) ratio in these assays is defined as the measured ELISA signal for a given patient sample, divided by that for the healthy control. The PCC Agent cocktail performed at least as well, and typically much better, than the standard chimeric protein antigen. The average S/N improvement by the cocktail PCC agent over the chimeric antigen was a factor of 2.5.

We then tested the PCC Agents for thermal stability. The PCC Agent cocktail component **(iii)** were synthesized at a large scale for an academic setting (∼7 mg, **Figure 3B**) and the lyophilized samples were stored under N_2_ at 25°C, 37°C, or 57°C for 58 days. The samples were then analyzed by HPLC to determine the presence of any degradation product. The traces of the peptide at each temperature are nearly identical, indicating little to no degeneration at these temperatures (**Figure 3B**). The performance of these stored PCC Agents was then also tested in an ELISA, with no detectable loss of performance (**Figure 3B**).

## Conclusion

We describe here a method for developing a PCC Agent cocktail to capture the diversity of human antibodies generated in response to an infectious agent. We demonstrated the successful application of this method for HIV-1 diagnostics by producing a cocktail of three PCC Agents that detected the presence of anti-HIV antibodies in clinical samples with a significantly enhanced signal-to-noise relative to the standard, recombinant protein-based chimeric antigen. In a recent report, an antigenic peptide cocktail comprised of synthetic peptides derived directly from gp120/V3-I (HIV-1 Indian isolate), gp41 (HIV-1), and gp36 (HIV-2), as well as the recombinant protein rp24 (HIV-1) was shown to also provide superior performance relative to the chimeric antigen [19]. This points to the possibility that expanding the current approach by developing multiple cocktails of PCC Agents, each directed against a distinct HIV epitope, would likely provide superior performance to that reported here. The strategy presented provides a promising approach for developing assays for detecting the immune response to other infectious agents, especially where challenges associated with the polyclonal nature of a humoral immune response can compromise assay sensitivity.

## Supporting Information

Materials and Methods S1 Detailed experimental protocols.
(DOCX)

Figure S1 **Differential detection of 3D6 and 4B3 by anchor ligands.** Relative affinities of A21 and A22 for 3D6 and 4B3 were determined by sandwich ELISA. Biotinylated anchor ligands A21 and A22 were immobilized on streptavidin-coated 96-well plated at a concentration of 100 nM, and incubated with the solutions of target anti-HIV antibodies 3D6 and 4B3 at 100 nM in TBS. Captured antibody was detected by peroxidase-conjugated anti-human IgG antibody.
(TIF)

Figure S2 **Apparent affinity of A21 and biligands directed against 3D6 as determined by SPR. A.** Sensorgram and 1^st^ order Hill fit to affinity data for A21. **B.** Sensorgram and 1^st^ order Hill fit to affinity data for A21-nidnG **(i)**. **C.** Sensorgram and 1^st^ order Hill fit to affinity data for A21-hnpfk **(ii)**.
(TIF)

Figure S3 **Apparent affinity of A22 and biligand directed against 4B3 as determined by SPR. A.** Sensorgram and 1^st^ order Hill fit to affinity data for A22. **B.** Sensorgram and 1^st^ order Hill fit to affinity data for A22-eihny **(iii)**.
(TIF)

Figure S4 **Performance of PCC agent cocktail to detect 3D6 and 4B3 from human serum.** Comparative performance of the PCC Agent cocktail versus the original gp41 epitope A21 and the commercial chimeric protein antigen was tested by a sandwich ELISA. Target antibodies 3D6 and 4B3 (4 nM each) were both spiked into diluted, HIV-free human serum (1% v/v in TBS), and captured antibody was detected by peroxidase-conjugated anti-human IgG antibody.
(TIF)

Table S1 **Biligand screen results.** List of pentapeptide “hits” from OBOC biligand screens performed with A21/3D6 and A22/4B3. The selected secondary ligands corresponding to **(i)**, **(ii)**, and **(iii)** are in bold.
(TIF)
