# Supplementary material for: A Cocktail of Thermally Stable, Chemically Synthesized Capture Agents for the Efficient Detection of Anti-Gp41 Antibodies from Human Sera
Source: PLoS One. 2013 Oct 7;8(10):e76224. doi: 10.1371/journal.pone.0076224 (PMC3792125; doi:10.1371/journal.pone.0076224)
Supplement: Materials and Methods S1 — Detailed experimental protocols. (DOCX) [file pone.0076224.s001.docx]

**Materials and Methods S1**

***Anchor Synthesis***

*A21 and A22*

The anchor peptides were synthesized C → N on 200-300 mgs of Rink amide resin (AnaSpec, Fremont, CA) with a Titan peptide synthesizer using standard Fmoc chemistry. The synthesis used Fmoc and side chain protected L amino acids (Aaptec, Louisville, KY), Fmoc protected propargylglycine (Pra) (Aaptec, Louisville, KY), PEG_5_ (EMD Millipore, Germany), and biotin (Sigma-Aldrich, St. Louis, MO). The finished peptides were side-chain deprotected and cleaved from resin in a mixture of 95% v/v trifluoroacetic acid (TFA) (Sigma-Aldrich, St. Louis, MO), 5% v/v dH_2_O, and 5% triethylsilane (TES) (Sigma-Aldrich, St. Louis, MO), and precipitated in diethyl either. Cu(Phen)_3_ stock solution was prepared by mixing 150 mM Cu(II) sulfate pentahydrate (Sigma-Aldrich, St. Louis, MO) and 500 mM 1,10-phenanthroline (Sigma-Aldrich, St. Louis, MO) in  4:1 H_2_O:Ethanol. The ether-precipitated ligands were cyclized by stirring the cleaved peptides dissolved in ~5 mL acetonitrile (ACN) overnight with 1 mL of the Cu(Phen)_3_ stock solution. The cyclization mixtures were lyophilized, and the final peptide products were purified using reverse phase (RP) HPLC. MALDI-TOF MS: A21: Expected mass [M+H]^+^ = 2006.96, Observed mass [M+H]^+^ = 2006.24. A22: Expected mass [M+H]^+^ = 1892.89, Observed mass [M+H]^+^ = 1892.85

***OBOC Screens***

All screens used naïve one-bead-one-compound (OBOC) D-pentapeptide libraries on TentaGel resin (TG) (Rapp Polymere, Tuebingen, Germany) of the form NH_2_-Az_4_-X_1_X_2_X_3_X_4_X_5_-TG, excluding cysteine and methionine, where Az_4_ is L-azidolycine. Libraries were synthesized on a Titan peptide synthesizer (Aaptec, Louisville, KY) using a split/mix method, and couplings were done using standard Fmoc SPPS chemistry in *N*-methylpyrrolidone (NMP).

*A21 with 3D6*

Screening against 3D6 was carried out in four main steps: pre-clear, target screen, anti-screen, and product screen. ***Step 1 preclear****:* 400 mg of OBOC library was blocked in 5% w/v dry milk in Tris buffered saline (TBS) (25 mM Tris-HCl, 150 mM NaCl, 10 mM MgCl_2_, pH 7.6) for 2 hr at room temperature, and then incubated with 1:10,000 phosphatase-conjugated streptavidin (SA-AP, Promega, Madison, WI) in 0.5% w/v dry milk in TBS for 1 hr. The beads were washed in high salt buffer (25 mM Tris-HCl, 750 mM NaCl, 10 mM MgCl_2_, pH 7.6) for 1 hr, and then developed with BCIP/NBT (Promega, Madison, WI) in BCIP buffer (100 mM Tris-HCl, 150 mM NaCl, 1 mM MgCl_2_, pH 9). After 20 minutes, the reaction was quenched with conc. HCl, and the purple beads were discarded. The remaining library was decolorized in NMP, dried with methanol (MeOH) and dichloromethane (DCM), and then swelled and blocked in 5% w/v dry milk in TBS for 2 hr at room temperature. ***Step 2 target screen****:* 4 mL of a solution containing 11.4 µM A21 and 147 nM 3D6 in 0.5% w/v dry milk in TBS was added to the precleared OBOC library, and the *in situ* click reaction was allowed to proceed overnight at 4°C. Bound target was probed with 1:10,000 AP-conjugated rabbit anti-human IgG Fc (Thermo Scientific, Rockford, IL) in 0.5% w/v dry milk in TBS for 1 hr at room temperature. The beads were then washed in high salt buffer for 1 hr at room temperature, and developed with BCIP/NBT in BCIP buffer. The reaction was quenched after 20 minutes with conc. HCl, and the purple beads were retained and decolorized in NMP and dried with MeOH and DCM. The hit beads were then stripped of all bound protein by washing with 7.5 M Guanidine-HCl (pH 2) for 2 hr at room temperature, rinsed 10 times with water, and then blocked in 5% w/v dry milk in TBS for 2 hr at room temperature. ***Step 3 anti-screen:*** A solution of 1% v/v human serum (Omega Scientific, Tarzana, CA) in TBS was incubated with the hit beads from Step 2 for 1 hr at room temperature. Off-target human antibodies bound to the beads were probed with 1:10,000 AP-conjugated rabbit anti-human IgG Fc in 0.5% w/v dry milk in TBS for 1 hr at room temperature. The beads were then washed in high salt buffer for 1 hr, and developed with BCIP/NBT in BCIP buffer. The reaction was quenched after 20 minutes with conc. HCl, and the purple beads were discarded. The remaining beads were decolorized in NMP and dried with MeOH and DCM. The anti-screened beads were swelled in water and washed with Guanidine-HCl for 2 hr at room temperature, rinsed 10 times with water, and then blocked in 5% w/v dry milk in TBS for 2 hr at room temperature. ***Step 4 product screen****:* The beads were incubated for 1 hr at room temperature with 1:10,000 SA-AP in 0.5% w/v dry milk in TBS, washed for 1 hr with high salt buffer, and then developed with BCIP/NBT in BCIP buffer for 20 minutes. The reaction was quenched with conc. HCl, and the purple beads were retained and sequenced using a Procise Protein Sequencing System (Applied Biosystems, Kingston, RI).

*A22 with 4B3*

Screening against 4B3 was carried out similarly to that described for 3D6, with two exceptions. In ***Step 2***, the *in situ* click reaction solution contained 10 µM A22 and 430 nM 4B3 in 0.5% w/v dry milk in TBS. In ***Step 3***, the beads were anti-screened against 0.1% v/v human serum in TBS spiked with 430 nM 3D6.

***Biligand Synthesis***

*Pra-OtBu*

The C-terminally protected Pra molecule used in the synthesis of **(i)** and **(ii)** was made by refluxing Fmoc-L-Pra-OH (Aaptec, Louisville, KY) with tert-butyl-tricholroacetamide (Sigma-Aldrich, St. Louis, MO) at 50°C for 3 hr in DCM. The formed product was separated from unreacted species on a silica flash column in DCM.

*Peptides (i) and (ii)*

The secondary ligands including N-terminal Az_4_ for **(i)** and **(ii)** were synthesized C → N on 200-300 mgs of Rink amide resin with a Titan peptide synthesizer using standard Fmoc chemistry. The synthesis used Fmoc and side chain protected D amino acids and L-Az_4_ (Aaptec, Louisville, KY). Fmoc- and C-terminally protected Pra (Pra-OtBu) was clicked on-bead to the azide side chain of Az_4_ by adding equal molar amounts of Pra-OtBu, Cu(I) iodide (Sigma-Aldrich, St. Louis, MO), and L-ascorbic acid (Sigma-Aldrich, St. Louis, MO), in 2x excess to the azide on bead. The click mixture was agitated in 20% v/v piperidine in NMP for 3 hr at room temperature, and the copper was chelated by repeated washing with 10% w/v sodium diethylduthiocarbamate trihydrate (Sigma-Aldrich, St. Louis, MO) and 10% v/v *N,N*-diisopropylethylamine (DIEA ) (Sigma-Aldrich, St. Louis, MO) in NMP until the beads were clear. The Fmoc deprotected Pra was then the starting point for the remainder of the anchor component of each ligand, including N-terminal PEG_5_ and biotin. The anchor components were synthesized using standard Fmoc chemistry with protected L amino acids. The finished peptides were side-chain deprotected and cleaved from resin in 95:5:5 TFA:dH_2_O:TES, then precipitated in diethyl either. The ligands were cyclized by stirring the cleaved peptides dissolved in ~5 mL ACN overnight with 1 mL of the Cu(phen)_3_ stock solution. The cyclization mixtures were lyophilized, and the final peptide products were purified using RP-HPLC. MALDI-TOF MS: **(i)**: Expected mass [M+H]^+^ = 2733.30, Observed mass [M+H]^+^ = 2733.64. **(ii)**: Expected mass [M+H]^+^ = 2843.40, Observed mass [M+H]^+^ = 2843.60.

*Peptide (iii)*

A22 was synthesized, cyclized, and purified as described above. The secondary ligand component including N-terminal Az_4_ for **(iii)** was synthesized C → N on Rink amide resin with a Titan peptide synthesizer using standard Fmoc chemistry. The synthesis used Fmoc and side chain protected D amino acids and L-Az_4_. Resulting secondary peptide was side-chain deprotected and cleaved from resin in 95:5:5 TFA:dH_2_O:TES, precipitated in diethyl either, and purified using RP-HPLC. The purified secondary peptide was clicked to A22 in solution by mixing with 2x molar excess of A22, 10x Cu(I) iodide, 50x L-ascorbic acid, and 4x Tris[(1-benzyl-1*H*-​1,2,3-​triazol-​4-​yl)​methyl]​amine (TBTA) (Sigma-Aldrich, St. Louis, MO) in 5:1 NMP:dH_2_O and stirring overnight at room temperature. The clicked biligand product was purified from the reaction mixture by RP-HPLC. Expected mass [M+H]^+^ = 2720.31, Observed mass [M+H]^+^ = 2720.5.

*Scaling up (iii)*

The secondary ligands including N-terminally Boc-protected Az_4_ for **(iii)** was synthesized C → N on Rink amide resin with a Titan peptide synthesizer using standard Fmoc chemistry. The synthesis used Fmoc and side chain protected D amino acids and L-Az_4_ (AnaSpec, Fremont, CA). Truncated A22 (Pra-ICTTA) was synthesized C → N on Sieber amide resin (ChemPep, Wellington, FL) with a Liberty 1 microwave peptide synthesizer (CEM, Matthews, NC) using standard Fmoc chemistry. The synthesis used Fmoc and side chain protected L amino acids and Fmoc-L-Pra. Fully protected truncated A22 was cleaved from the resin with 1% v/v TFA in DCM, and neutralized with DIEA. Solvent was removed using a rotary vacuum evaporator and the protected peptide was purified by RP-HPLC. Resulting truncated A22 was clicked on-bead to the secondary peptide through the azide side chain of Boc-Az_4_ by adding equal molar amount of the protected truncated A22, 2x molar excess of Cu(I) iodide, and 2x excess of L-ascorbic acid to the azide on bead. The click mixture was agitated in 20% v/v piperidine in NMP overnight at room temperature, and then the copper was chelated by repeated washing with 10% w/v sodium diethylduthiocarbamate trihydrate and 10% v/v DIEA in NMP until the beads were clear. The Fmoc deprotected Pra was then the starting point for the remainder of the anchor component, including N-terminal PEG_5_ and biotin (Biotin-PEG_5_-IWGCSGK). The anchor components were synthesized using standard Fmoc chemistry with protected L amino acids. The finished peptide was N-terminal and side-chain deprotected and cleaved from resin in 95:5:5 TFA:dH_2_O:TES, then precipitated in diethyl either. The ligand was cyclized by stirring the cleaved peptide dissolved in ~5 mL ACN overnight with 1 mL of the Cu(phen)_3_ stock solution. The cyclization mixtures were lyophilized, and the final peptide products were purified using RP-HPLC.

***Assays***

*Anchor binding ELISA*

Biotinylated anchor peptide was immobilized on streptavidin-coated 96-well plates (Thermo Scientific Pierce, Rockford, IL) at a concentration of 100 nM in TBS. The plates were blocked with 5% w/v dry milk in TBS, and then the anchor peptides were incubated with either 100 nM 3D6 or 4B3 in 0.5% w/v dry milk in TBS at room temperature for 1 hr. Bound antibody was probed with horseradish peroxidase (HRP)-conjugated mouse monoclonal antibody to human IgG Fc (Abcam, Cambridge, MA), diluted 1:10,000 in 0.5% w/v dry milk in TBS for 1 hr at room temperature. The colorimetric assay was developed with TMB substrate (KPL, Gaithersburg, MD), then quenched with 1 M H_2_SO_4_ and read at 450 nm.

*Surface Plasmon Resonance*

SPR experiments were performed on a Biacore T200 instrument. Biotinylated peptides were immobilized on streptavidin-coated sensor chips (GE Healthcare Biosciences, Pittsburg, PA) at R.U. values ranging from 7-12. 4B3 or 3D6 was flowed at various concentrations in 1x HBS-EP buffer (GE Healthcare Biosciences, Pittsburg, PA), and affinity curves were fit using the default settings of the Biacore evaluation software.

*Cocktail binding ELISA*

Recombinant multi-epitope chimeric HIV antigen (“chimera”) (BioLink International, Lisle, IL) was chemically biotinylated using ChromaLink Biotin Labeling Kit (Solulink, San Diego, CA) according to the manufacturer’s instructions, using 10x molar excess of the ChromaLink biotinylation reagent to the buffer-exchanged protein. Streptavidin-coated 96-well plates were saturated with the biotinylated biligand cocktail or chimera using 1 uM solutions in TBS. The plates were blocked with 5% w/v dry milk in TBS, and then the capture reagents were incubated with either 1% v/v human serum or a mixture of 4 nM each 3D6 and 4B3 spiked in 1% v/v human serum in TBS at room temperature for 1 hr. Bound antibody was probed with HPR-conjugated mouse monoclonal antibody to human IgG Fc, diluted 1:15,000 in 0.5% w/v dry milk in TBS for 1 hr at room temperature. The colorimetric assay was developed with TMB substrate, then quenched with 1 M H_2_SO_4_ and read at 450 nm (A450). The A450 for each sample is normalized against the A450 for the serum control, to yield a measurement of the signal-to-noise ratio of the assay.

*Patient serum ELISA*

Peripheral blood was obtained from HIV-1 infected patients at the University of California, Los Angeles (UCLA) Medical Center between July and August of 2012. Sera were stored at -80°C before subsequent analysis. Streptavidin-coated 96-well plates were saturated with the biotinylated biligand cocktail or chimera using 1 uM solutions in TBS. The plates were blocked with 5% w/v dry milk in TBS, and then the capture reagents were incubated with patient serum samples diluted to 1% v/v in TBS containing 0.1% w/v bovine serum albumin (BSA). Additionally, both cocktail and chimera capture reagents were incubated with 1% v/v commercial healthy human serum in TBS. Bound antibody was probed with HPR-conjugated mouse monoclonal antibody to human IgG Fc, diluted 1:10,000 in TBS with 0.1% w/v BSA. The colorimetric assay was developed with TMB substrate, then quenched with 1 M H_2_SO_4_ and read at 450 nm (A450). The A450 for each sample is normalized against the A450 for the healthy control, to yield a measurement of the signal-to-noise ratio of the assay.

*Stability assay*

Small amounts of **(iii)** were stored under N_2_ at 25°C, 37°C, or 57°C for 58 days. Samples were diluted in 1:1 ACN/H_2_O with 0.1% v/v TFA to an equal concentration determined by measuring the absorbance values at 280 nm using NanoDropspectrophotometer (Thermo Scientific, Waltham, MA) and resolved by analytical RP-HPLC. The remaining samples were lyophilized and used in a single point sandwich ELISA. Streptavidin-coated 96-well plates were saturated with the samples of **(iii)** recovered from the storage experiments using 1 uM solutions in TBS. The plates were blocked with 5% w/v dry milk in TBS, then incubated with either 1% v/v human serum or 10 nM 4B3 spiked in 1% v/v human serum in 0.5% w/v dry milk in TBS at room temperature for 1 hr. Bound antibody was probed with HPR-conjugated mouse monoclonal antibody to human IgG Fc diluted 1:15,000 in 0.5% w/v dry milk in TBS. The colorimetric assay was developed with TMB substrate, then quenched with 1 M H_2_SO_4_ and read at 450 nm.
