# Supplementary figures and images for: A Cocktail of Thermally Stable, Chemically Synthesized Capture Agents for the Efficient Detection of Anti-Gp41 Antibodies from Human Sera
Source: PLoS One. 2013 Oct 7;8(10):e76224. doi: 10.1371/journal.pone.0076224 (PMC3792125; doi:10.1371/journal.pone.0076224)

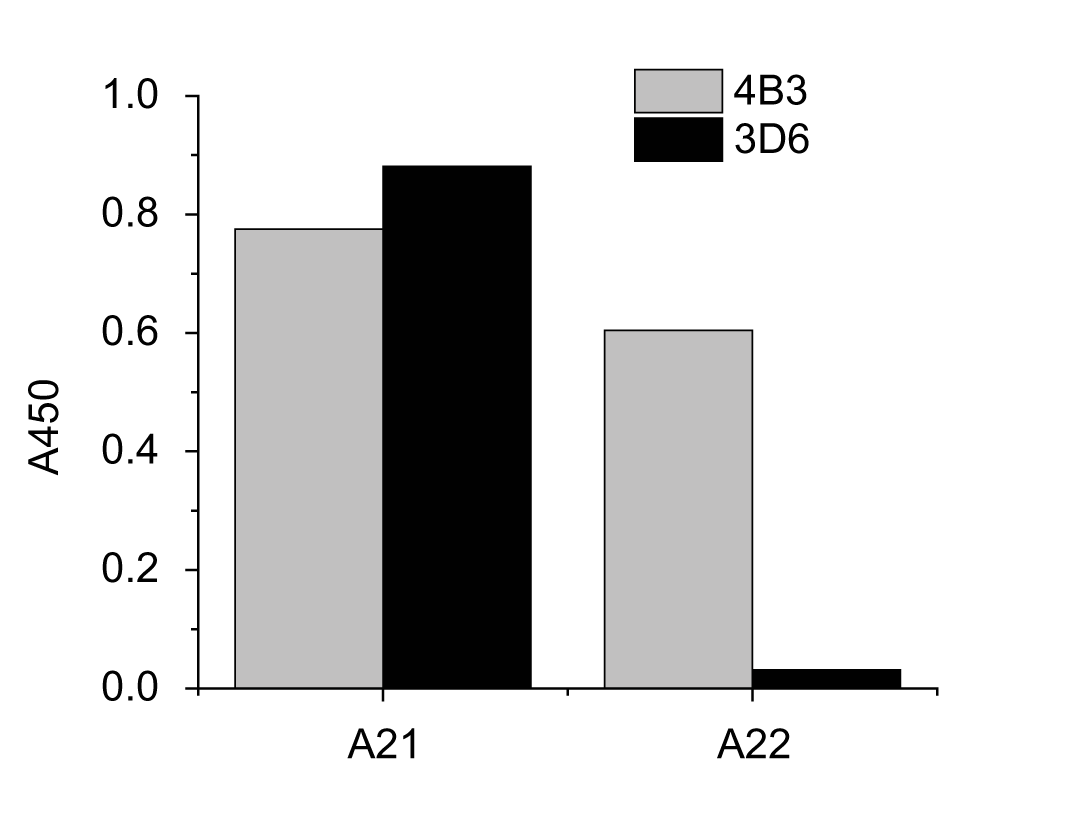

Supplement: Figure S1 — Differential detection of 3D6 and 4B3 by anchor ligands. Relative affinities of A21 and A22 for 3D6 and 4B3 were determined by sandwich ELISA. Biotinylated anchor ligands A21 and A22 were immobilized on streptavidin-coated 96-well plated at a concentration of 100 nM, and incubated with the solutions of target anti-HIV antibodies 3D6 and 4B3 at 100 nM in TBS. Captured antibody was detected by peroxidase-conjugated anti-human IgG antibody. (TIF) [file pone.0076224.s002.tif]

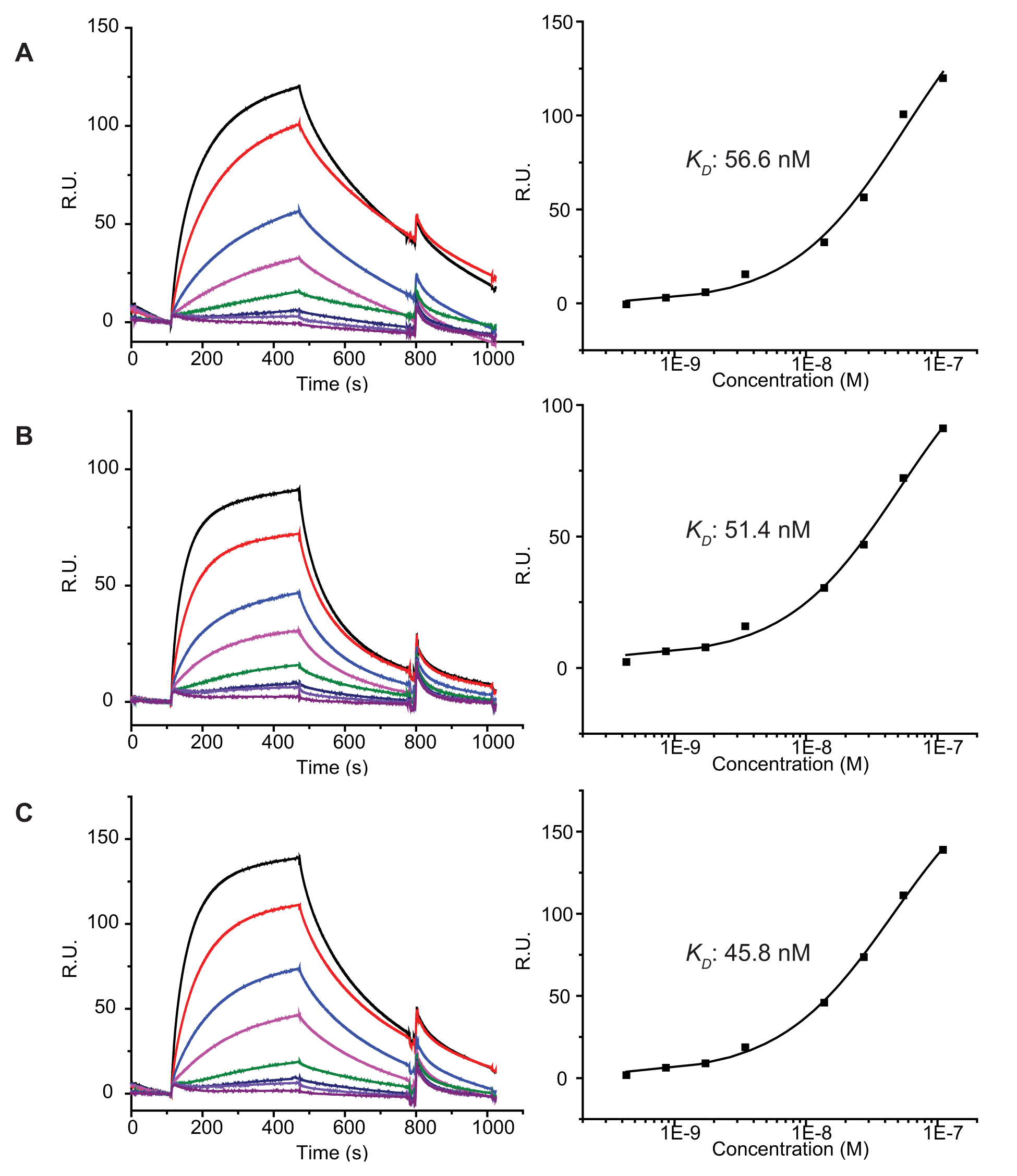

Supplement: Figure S2 — Apparent affinity of A21 and biligands directed against 3D6 as determined by SPR. A. Sensorgram and 1st order Hill fit to affinity data for A21. B. Sensorgram and 1st order Hill fit to affinity data for A21-nidnG (i). C. Sensorgram and 1st order Hill fit to affinity data for A21-hnpfk (ii). (TIF) [file pone.0076224.s003.tif]

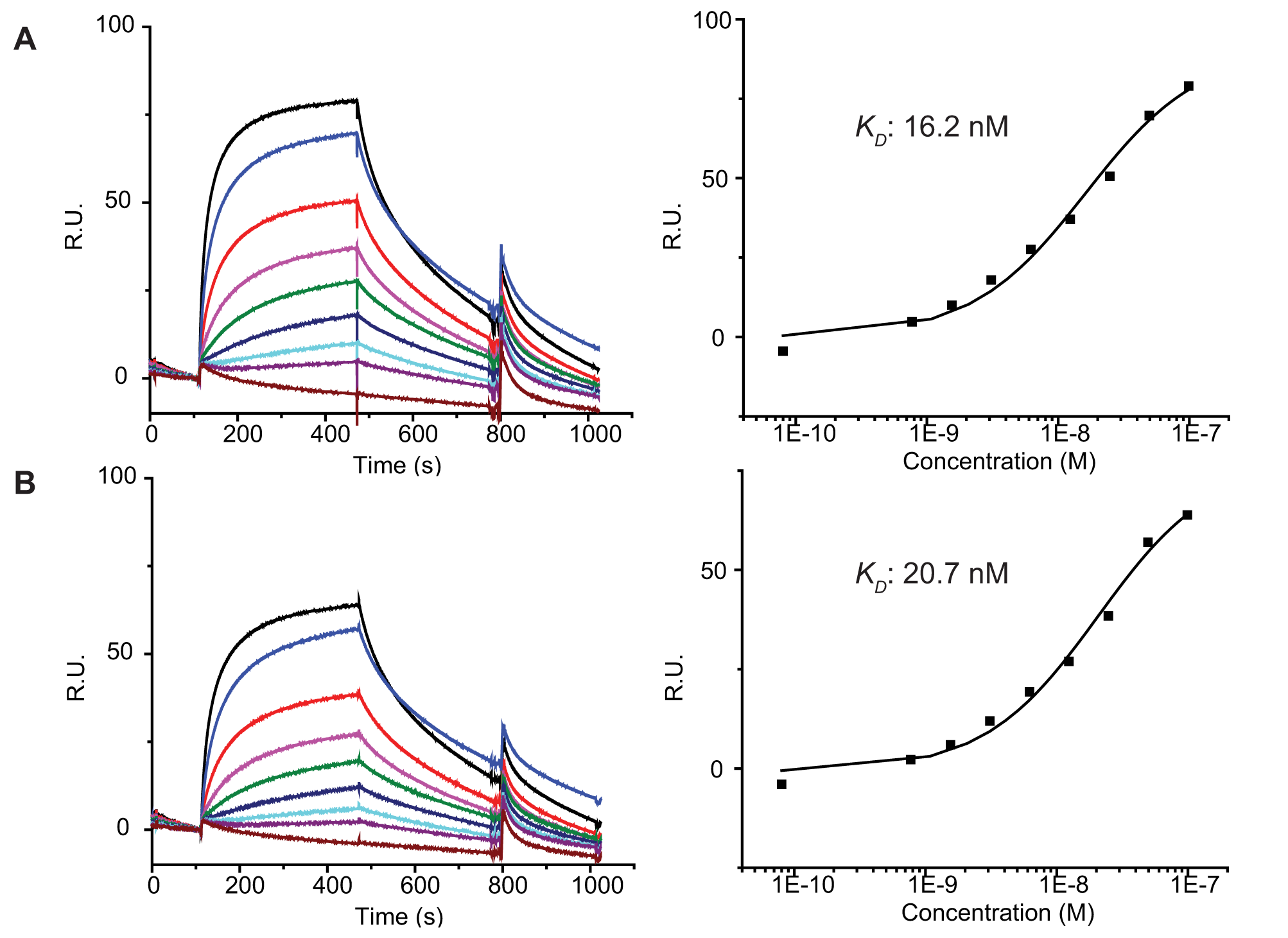

Supplement: Figure S3 — Apparent affinity of A22 and biligand directed against 4B3 as determined by SPR. A. Sensorgram and 1st order Hill fit to affinity data for A22. B. Sensorgram and 1st order Hill fit to affinity data for A22-eihny (iii). (TIF) [file pone.0076224.s004.tif]

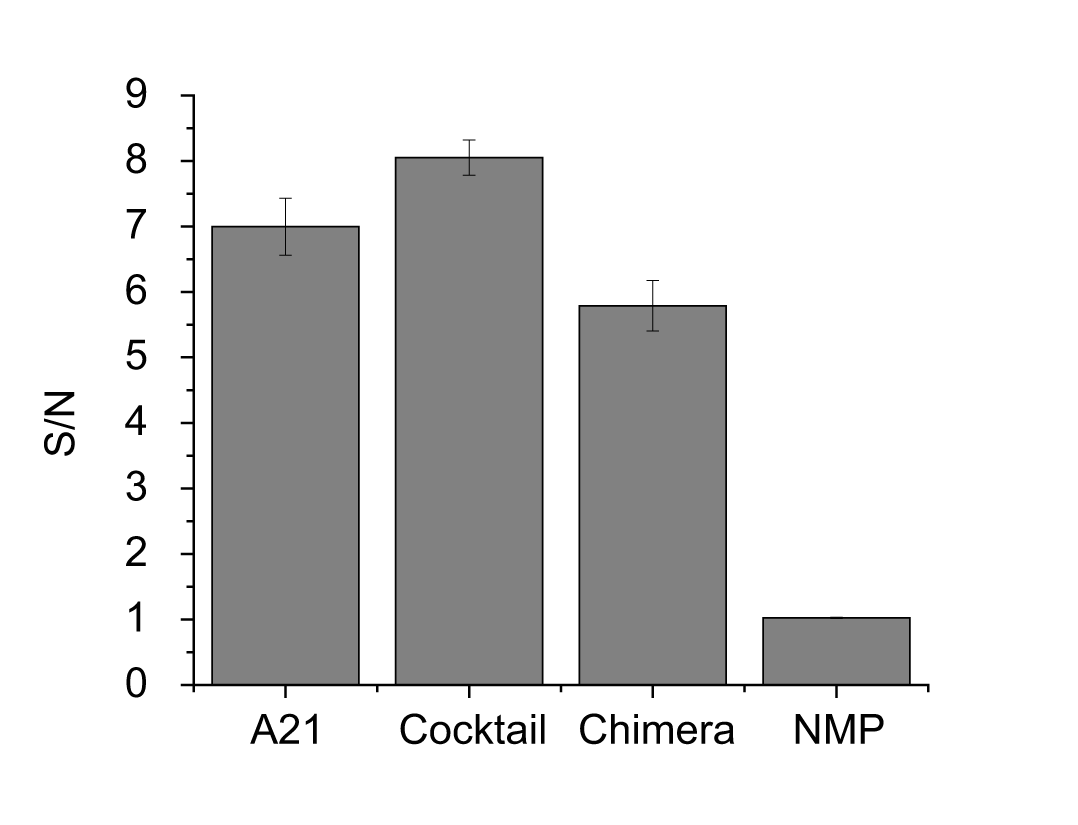

Supplement: Figure S4 — Performance of PCC agent cocktail to detect 3D6 and 4B3 from human serum. Comparative performance of the PCC Agent cocktail versus the original gp41 epitope A21 and the commercial chimeric protein antigen was tested by a sandwich ELISA. Target antibodies 3D6 and 4B3 (4 nM each) were both spiked into diluted, HIV-free human serum (1% v/v in TBS), and captured antibody was detected by peroxidase-conjugated anti-human IgG antibody. (TIF) [file pone.0076224.s005.tif]

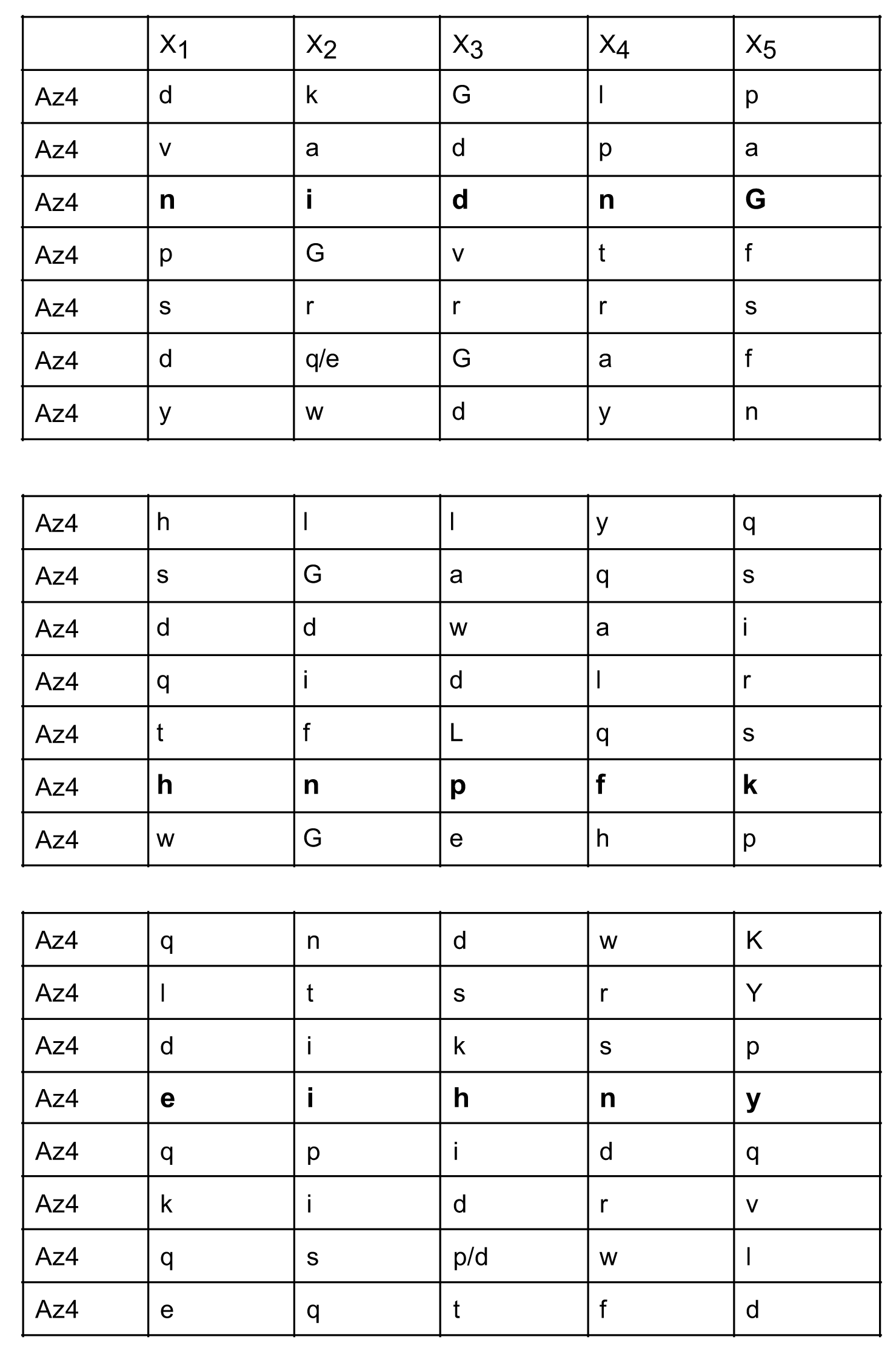

Supplement: Table S1 — Biligand screen results. List of pentapeptide “hits” from OBOC biligand screens performed with A21/3D6 and A22/4B3. The selected secondary ligands corresponding to (i), (ii), and (iii) are in bold. (TIF) [file pone.0076224.s006.tif]
